# The Presence and the Search Constructs of Meaning in Life in Suicidal Patients Attending a Psychiatric Emergency Department

**DOI:** 10.3389/fpsyt.2020.00327

**Published:** 2020-04-28

**Authors:** Alessandra Costanza, Marc Baertschi, Hélène Richard-Lepouriel, Kerstin Weber, Maurizio Pompili, Alessandra Canuto

**Affiliations:** ^1^Department of Psychiatry, Faculty of Medicine, University of Geneva (UNIGE), Geneva, Switzerland; ^2^Department of Psychiatry, ASO Santi Antonio e Biagio e Cesare Arrigo Hospital, Alessandria, Italy; ^3^Service of General Psychiatry and Psychotherapy, Nant Foundation, Montreux, Switzerland; ^4^Service of Psychiatric Specialties, Department of Psychiatry, Geneva University Hospitals, Geneva, Switzerland; ^5^Division of Institutional Measures, Medical Direction, Geneva University Hospitals, Geneva, Switzerland; ^6^Department of Neurosciences, Mental Health and Sensory Organs, Suicide Prevention Center, Sant’Andrea Hospital, Sapienza University of Rome, Rome, Italy

**Keywords:** suicide, suicidal behavior, suicidal ideation, suicide attempt, Meaning in Life, protective factors

## Abstract

Meaning in Life (MiL) is considered protective against suicidal behavior (SB). However, few studies specifically addressed the role of the constructs, “presence of MiL” and “search for MiL,” and their dynamic interplay. In this cross-sectional study of patients with SB (N = 199) visiting a psychiatric Emergency Department for either suicidal ideation (SI) or suicide attempt (SA), we pursued the following objectives: 1) to explore the relationship between the two constructs; 2) to verify the protective value of presence of MiL on SB; and 3) to assess the influence of search for MiL on the relationship between presence of MiL and SI. The two constructs were found to be independent of one another. Higher presence of MiL was globally associated with lower SB levels, particularly SI. Search for MiL was not related to SB and did not moderate the relationship between presence of MiL and SI. In conclusion, formal support for the role of presence of MiL against SB in a psychiatric sample was demonstrated. These findings, with a view toward refinement of SB risk assessment and new psychotherapeutic approaches, may lead to an enrichment of the dialogue with suicidal patients to help alleviate their unbearable suffering. Our conclusions must be replicated in psychiatric clinical populations in settings other than a psychiatric ED and by using a longitudinal prospective and case-control study design.

## Introduction

The presence of Meaning in Life (MiL) has long been associated with protection against suicide, as initially observed in prisoners of Nazi concentration camps (1, 2). More recently, MiL has been conceptualized as a psychological dimension (3–6), and a differentiation between “presence of” and “search for” MiL constructs has been proposed (7, 8). The former construct has been associated with benefits in various functioning aspects, including adaptive resources, overall psychological well-being, and positive affects (9). The impact of the latter construct has been more debated: some have considered it to be a primary human endeavor aimed at the challenges of life experiences to avoid pathological responses to negative situations (2, 10, 11), whereas others have associated its emergence with MiL dysfunction (12, 13). It has been proposed that the search for MiL is influenced by adaptive or non-adaptive aspects depending on the motivation, personality, and cognitive style of the individual (5, 14).

To our knowledge, only four studies have investigated the roles of both constructs of MiL in the development of suicidal behavior (SB) [(15–18); for a review, see (19)]. These studies were performed in undergraduate students (16), military personnel and veterans (15, 16, 18), and HIV-positive patients (17). All of these studies have reported that the presence of MiL has a protective function on SB, directly or through mediation-moderation models. Data describing search for MiL were less consistent but generally not associated with protection against SB (15–19). Although only a limited number of studies have reported on MiL in relation to SB, more research is available on MiL in those who live with chronic pain. For example, in a typological study among patients with chronic pain, five MiL profiles emerged that resulted from five different combinations of the two MiL constructs (20, 21). Both constructs and the related MiL profile were found to be fairly stable over time, leading to the hypothesis that they can reflect a trait aspect rather than a state aspect of the individual functioning (20, 21). However, the included patients were selected individual members of a pain management organization who had reported a relatively longer duration of chronic pain. It was suggested that this group had possibly completed the psychological acceptation and integration process of their pathological condition and were spared from experiencing it as a destabilizing event at the time of the study. Although the MiL profiles were not studied here in relation to SB, the authors proposed replicating such analyses in a sample of newly diagnosed chronic pain patients to possibly observe more changes in maintaining, losing, or restoring their MiL (20, 21) In patients suffering from chronic pain, a protective role of the presence of MiL against suicidal ideation was postulated (22). These data suggest that clinical interventions that take into account both of the constructs could enrich the encounter and the dialogue with the suicidal patient, to refine the SB risk assessment, and to offer new approaches for psychotherapeutic interventions (23–26).

The limited amount of research conducted in this area suggests that further investigation is necessary to draw definitive conclusions on the clinical applicability of MiL in SB management. With this study, we aimed to investigate the impact of both MiL constructs in a clinical population composed of patients visiting the psychiatric Emergency Department (ED) of a general hospital for SB, namely suicidal ideation (SI) or suicide attempt (SA). To this end, we pursued the following objectives: 1) to explore the relationship between the two constructs; 2) to verify the protective value of presence of MiL on SB; and 3) to assess the influence of search for MiL on the relationship between presence of MiL and SI. Based on the aforementioned published studies, we hypothesized the following: 1) that the two constructs are independent of one another; 2) that the presence of MiL, but not search for MiL, has a protective impact on both SI and SA; and 3) search for MiL will have no impact on the relationship between presence of MiL and SI.

## Methods

### Procedure

Every person referred to the adult division of the psychiatric EDs of the Geneva University Hospitals, Switzerland, from October 1, 2014, to February 1, 2016, for SB and not requiring admission to an inpatient somatic unit was offered the opportunity to participate in this study. Inclusion criteria were the occurrence of SI or SA, being aged 16 years (the minimal age to be admitted in adult healthcare departments in Geneva) or more, giving written consent, and completing the study procedure within 7 days following inclusion. We used the nomenclature proposed by Posner (27), which defines SI as “passive thoughts about wanting to be dead or active thoughts about killing oneself, not accompanied by preparatory behavior” and SA as “a potentially self-injurious behavior, associated with at least some intent to die, as a result of the act. Evidence that the individual intended to kill him/herself, at least to some degree, can be explicit or inferred from the behavior or circumstance. A suicide attempt may or may not result in actual injury” (p. 1037). Participants answered inquiries regarding their sociodemographic situations as well as a series of questionnaires, some administered by the psychiatrist in charge of the study inclusion and some self-reported. Other aspects of this global project on suicide, not relevant to the present study, have resulted in five publications (28–32). This study was approved by the local Research Ethics Committee under the registration number 14-168.

### Participants

129 of the 368 individuals offered to participate in this study declined for several reasons (e.g., lack of concentration, or interest, or insufficient fluency in the French language), and 40 were excluded by the psychiatrist in charge because of poor quality of data (e.g., repeated patterns in answers or questionnaires left blank) or procedural shortcomings (e.g., lack of a signed consent form). The final study group comprised of 199 participants, aged 33.3 ± 14.5 years (range, 16–82 years). The majority had a psychiatric diagnosis according to the Mini-International Neuropsychiatric Interview (33). The most prevalent diagnoses were episodes of major depression (69.8%), alcohol dependence (17.1%), and non-alcohol substance dependence (10.1%). Additional sociodemographic characteristics are summarized in **Table 1**.

**Table 1:** Psychosocial characteristics (*N* = 199).

		*n*	Percentage
Sex	Female	120	60.3
	Male	79	39.7
Age group	<20 years	42	21.1
	20 to <30 years	52	26.1
	30 to <40 years	39	19.6
	40 to <50 years	32	16.1
	50 to 60 years	26	13.1
	>60 years	8	4.0
Citizenship	Swiss	115	57.8
	Non-Swiss	84	42.2
Marital status	In a relationship	68	34.2
	Single	131	65.8
Children	Yes	78	39.2
	No	121	60.8
Professional status	Employed/Student	115	57.8
	No activity	84	42.2
Perceived wealth status	High (categories 1–3)	36	18.1
	Low (categories 4–6)	163	81.9
Inclusion criterion	Suicidal ideation	131	65.8
	Suicide attempt	68	34.2
Psychiatric diagnosis	Yes	177	88.9
	No	22	11.1

Perceived wealth status was assessed by asking participants to rank themselves in one among six categories, from 1 (rich) to 6 (poor) on the basis of a short-written description.

### Instruments

SI was evaluated using the Scale for Suicide Ideation (SSI) (34), a self-administered questionnaire of 19 items rated on a three-point Likert scale (total score range, 0–38) with statements that varied from one item to another. The SSI has been validated in French (35, 36). Internal consistency was good within this data set (α = 0.844, 95% CI [0.811, 0.874]).

The Meaning in Life Questionnaire (MLQ) (8) is a self-reported 10-item inventory measuring the presence of MiL (5 items) and search for MiL (5 items) constructs. Each item was rated on a 7-point Likert scale from “absolutely true” to “absolutely untrue,” leading to a score range of 5 to 35 for each subscale. The MLQ has been translated into French and is available on the author’s website (http://www.michaelfsteger.com). However, to our knowledge, no formal validation study of the French version has been conducted. In our sample, internal consistency was good for the presence of MiL construct subscale (α = 0.802, 95% CI [0.755, 0.842]) and for search for MiL construct subscale (α = 0.877, 95% CI [0.848, 0.902]).

### Statistical Analyses

To address the first objective of this study, bivariate Pearson’s and point-biserial correlation analyses were conducted to explore the relationships between the variables of interest and sociodemographic parameters. To address the second objective, a 2-step hierarchical multiple linear regression model and a two-step logistic regression model were used. Finally, the third objective was pursued using a multiple linear regression model including the creation of an interaction variable.

All statistical analyses were conducted using Statistica version 13.0 (StatsSoft Inc., Tulsa, OK, USA), and a threshold of *p* ≤ 0.05 was considered to be statistically significant.

## Results

### Relationships Between the Variables of Interest

Bivariate Pearson’s correlations were computed for the presence of MiL and search for MiL construct subscales of the MLQ for the entire sample and separately for those with SAs and SI, as grouping variables. The subscales were not significantly correlated in the whole sample (*M*_Presence_ = 18.357 ± 7.065, *M*_Search_ = 24.955 ± 7.161, *r* = −0.015, *p* = 0.831), nor for those with SAs (*M*_Presence_ = 20.353 ± 7.011, *M*_Search_ = 24.456 ± 7.170, *r* = −0.181, *p* = 0.139), nor with SI (*M*_Presence_ = 17.321 ± 6.893, *M*_Search_ = 25.214 ± 7.170, *r* = 0.087, *p* =.321). Additionally, a point-biserial correlation showed that participants recruited for a SA had higher presence of MiL construct scores compared to those with SI only (SI = 0, SA = 1, *r_pb_* =.204, *p* < 0.05). No significant correlation was found between search construct subscale and SI/SA. Finally, unlike the search for MiL construct, the presence of MiL construct was negatively correlated with the SSI score (*r* = −0.485, *p* < 0.05).

We then measured the associations between the two constructs and several sociodemographic variables (i.e., age, children, marital status, professional situation, and perceived wealth). A bivariate Pearson’s correlation showed that the presence of MiL construct was positively correlated with age (*r* = 0.225, *p* < 0.05), while point-biserial correlations underscored a positive relationship between the presence of MiL construct and having at least one child (when no children = 0 and at least one child =1, *r_pb_* = 0.334, *p* < 0.05). Also, a positive relationship was found between the presence of MiL construct and being in a couple relationship (when single = 0 and in a relationship = 1, *r_pb_* = 0.236, *p* < 0.05). No sociodemographic variable was significantly associated with the search for MiL construct.

Next, we explored the relationships between the two constructs and the psychiatric diagnoses defined by the Mini-International Neuropsychiatric Interview in conducting point-biserial correlations (0 = no diagnosis, 1 = diagnosis). There was a negative association between the presence of MiL construct and having a psychiatric diagnosis (*r_pb_* = −0.157, *p* < 0.05). Similarly, a negative association was found between the presence of MiL construct and a diagnosis of past major depressive disorder (*r_pb_* = −0.155, *p* < 0.05). Finally, while participants with current social phobias scored significantly higher on the search construct scale (*r_pb_* = 0.149, *p* < 0.05), this result should be interpreted with caution since only 5 participants (2.5%) had this diagnosis.

### Presence of MiL Construct as a Predictor of SB

A two-step multiple linear regression equation was built using the SSI score as the dependent variable. In the first step, we used the sociodemographic parameters that showed positive correlations with the presence of MiL construct, namely age, having children, and relationship status. As summarized in **Table 2**, this initial model significantly predicted the SSI score (*F* [3, 195] = 4.129, *p* = 0.007) and accounted for 4.5% of the global variance in SI as measured by the adjusted *R*^2^. The only sociodemographic variable predicting the SSI score was marital status (*b* = −2.506, *p* = 0.050), suggesting that being in a relationship reduces the likelihood of developing SI. In the second step, we added the presence of MiL construct as a predictor of the dependent variable. As shown in **Table 2**, this model predicted the SSI score (*F* [4, 194] = 15.711, *p* < 0.001) and accounted for 22.9% of the total variance. The presence of MiL construct was the only single variable that had a significant effect on SI (*b* = −0.530, *p* < 0.001).

**Table 2:** Two-step hierarchical multiple regression equation with sociodemographic variables and presence of Meaning in Life (MiL) construct predicting suicidal ideation (*N* = 199).

Predictors entered as a set	*F*	*df*	*R^2^*	Adjusted *R^2^*	*b*	*t*	*p*
1	4.129	3, 195	0.060	0.045			**0.007**
Age					−0.000	−0.008	0.994
Children					−2.496	−1.799	0.074
Marital status					−2.506	−1.971	**0.050**
2	15.711	4, 194	0.245	0.229			**<0.001**
Age					0.021	0.520	0.604
Children					−0.715	−0.562	0.575
Marital status					−1.436	−1.245	0.215
MLQ presence of MiL construct					−0.530	−6.892	**<0.001**

In bold, p values significant at the ≤ 0.05 threshold. Coding for dummy variables are as follows. Children: 0 (no children) or 1 (at least one child); marital situation: 0 (single) or 1 (in a relationship).

We then built a logistic regression model using a criterion variable that dichotomized participants incorporated in the study for SI only (coded 0) from those included in the study for an SA (coded 1). As before, we first used the sociodemographic variables that showed positive correlations with the presence of MiL construct and in this case found that the model accounted for a small proportion of the variance in the study inclusion criterion (deviance = 252.839, df = 195, Cox-Snell *R^2^* = 0.014, Nagelkerke *R^2^* = 0.019), and the odds ratio (OR) of each sociodemographic variable did not predict the dependent variable at our significance threshold. Thereafter, we added the presence of MiL construct as predictor in a second model, and it accounted for a larger proportion of the variance in the dependent variable (deviance = 246.009, df = 194, Cox-Snell *R^2^* = 0.047, Nagelkerke *R^2^* = 0.065). In addition, the presence of MiL construct predicted the probability to be included in the study with an SA rather than with SI only (OR = 1.062, 95% CI [1.014, 1.112], *p* = 0.010).

### Search for MIL Construct as a Moderator of Between Presence of MiL Construct and SI

To assess the moderating effect of the search for MiL construct on the relationship between the presence of MiL construct and SI as measured by the SSI score, a multiple linear regression analysis was designed with the SSI score as the criterion variable and the two constructs as predictors. In addition, we used an interaction variable between the two constructs. Because of multicollinearity effects, results for the two constructs were centered around their means, and the interaction variable was created from these mean-centered variables.

As shown in **Table 3**, this model significantly predicted the SSI score, (*F* [3, 195] = 21.094, *p* < 0.001), and explained 23.3% of its total variance (adjusted *R^2^*). Although the presence of the MiL construct had a protective effect on SI (*b* = −0.538, *p* < 0.001), neither search construct nor the interaction variable between presence of MiL construct and search construct had a significant effect on the dependent variable.

**Table 3:** Multiple regression equation with presence and search constructs of Meaning in Life (MiL) predicting suicidal ideation (*N* = 199).

Predictors entered as a set	*F*	df	*R^2^*	Adjusted *R^2^*	*b*	*t*	*p*
1	21.094	3, 195	0.245	0.233			**<0.001**
Presence of MiL construct					−0.538	−7.386	**<0.001**
Search for MiL construct					0.030	0.415	0.678
Presence of MiL construct x search construct					0.011	1.457	0.147

In bold, p values significant at the ≤ 0.05 threshold.

## Discussion

This study investigated the role of the two MiL constructs in a clinical population of patients attending a psychiatric ED for SB, namely SI and SA.

Regarding our first hypothesis, our data confirmed that the two MiL constructs were independent of one another, in line with previous research (8, 14). Although not significant, the relationship between the two constructs in our study was negative, which corresponds to other studies that have consistently shown this trend, sometimes significant when the effect size was small (14, 37). The independence of the two constructs can create only an apparent conceptual paradox, which has been thoroughly discussed (8). Previous work has shown that because they are independent, these constructs can be assessed separately. In particular, this independence can override the debate over whether a beneficial versus dysfunctional role for the search for MiL construct and its dynamic interplay with the presence of MiL construct *via* a greater theoretical flexibility. For instance, it can allow the identification and distinction of individuals who feel great meaningfulness yet are still engaged in seeking life’s meaning and contrast them with those who feel the same meaningfulness but are not engaged in further search for meaning (8).

We also investigated possible relationships between the constructs and other variables of interest, such as sociodemographic variables. To our knowledge, this kind of data has not yet been reported. The characteristics of our sample suggested that it was representative of individuals visiting ED for a non-lethal suicidal event with a predominance the female sex (38–42), a mean adult age less than 50 years (38, 41, 43, 44), and the presence of a psychiatric disorder in most participants (39, 44, 45). Although the search for MiL construct was not associated with any sociodemographic variable, the presence of MiL construct correlated positively with factors known to protect against SB, such as middle adult age, having at least one child, and being in a couple relationship (46, 47). Similarly, we found a negative correlation between the presence of MiL construct and having a psychiatric diagnosis, and this correlation notably applied to a diagnosis of a past major depressive disorder. A psychiatric diagnosis is among the most significant risk factors for suicide (46, 47). Thus, the presence of MiL construct could be considered a protective factor against the development of psychiatric disorders in general, and suicide in particular. Taken together, these findings suggest that the presence of MiL construct is not an innate attribute and does not boil down to a personality trait. Rather, it is likely a dynamic construct requiring time for maturation, as implied by its positive association with age. The presence of MiL construct also appears to be dependent on significant life events such as childbirth or marriage. Because of the cross-sectional design of this study, we were unable to confirm these assumptions.

The typological approach utilized in the aforementioned chronic pain studies relates to the discussion of our first hypothesis (i.e., the two constructs are independent of one another) by providing a research path that reinforces the proven independence of the two MiL constructs. It follows that a more nuanced and dynamic description of the individual’s personal attitude toward MiL can be generated through investigation of the constructs in the following combinations: 1) high presence of MiL-high search for MiL (i.e., patients experiencing MiL and searching for MiL); 2) high presence of MiL-low search for MiL (i.e., patients experiencing MiL but not searching for MiL); 3) moderate presence of MiL-moderate search for MiL (i.e., patients moderately experiencing MiL and searching for MiL); 4) low presence of MiL-low search for MiL (i.e., patients not experiencing MiL and not engaged in any search for MiL); and 5) low presence of MiL-high search for MiL (i.e., patients not experiencing MiL but engaged in search for MiL) (20, 21). These distinctions, which can also be used in relation to chronic pain or SB, are in line with the possibility of overriding the debate over a beneficial versus dysfunctional role for the search for MiL construct by utilizing a more flexible theoretical perspective (14).

With regard to our second hypothesis, that is that the presence of MiL construct has a protective impact on both SI and SA, we found that higher presence of MiL was associated with lower SI. No significant correlation was found between the search for MiL construct and SI/SA. Previous studies that have addressed this issue (15–18) reported similar results in that the presence of MiL construct was negatively associated with SB, particularly with SI. This also included a protective function of the presence of MiL construct on SA (16), but this could not be confirmed in our study since the absence of a non-suicidal control group negated a definitive conclusion. Similarly, we did not find a significant relationship between the search for MiL construct and SB, contrary to research that noted a protective effect on SI (16) and, in contrast, positive relationships with SI and SA (15). With reference to the described MiL profiles in patients with chronic pain, it was found that they were associated with a unique adjustment outcome that was more favorable for those that scored high in presence of MiL (20, 21). As previously specified, however, their impact on SB was not examined.

Somewhat counterintuitively, the presence of MiL construct predicted the probability of being included in the study with an SA rather than with SI only. To our knowledge, this is the first study to report this finding. In our sample population, participants with an SA had lower SI than those included with SI only, possibly implying that the psychological process that unfolds after an SA can have mitigating effects on SI (30). Because the correlation between the presence of MiL construct and SI, as measured using the SSI, was strongly negative (*r* = −0.485), we can hypothesize that this MiL construct was influenced by the latter psychological process on SI and, consequently, possibly more so before rather than after an SA. Unfortunately, the data providing information on the attitudes and reactions of individuals following an SA are limited, but one study found that a process of awareness of life responsibilities occurred at an initial stage (48). Similarly, it was pointed out that patients undergo a change in self-perception toward redefinition of self-image, life goals, and coping strategies and that this process should be undertaken in consideration of significant others (49). In addition, patients having made an SA used paired testimony to develop a personal sense of hope (50). Though the temporal nature of our study makes it unlikely that participants benefited from the testimonies of others at the time of questionnaire completion, one can speculate whether a search for hope was already instilled at an unconscious level following their SA. Overall, these findings suggest that an SA is followed by increased consideration of existential factors in life and, thus, can provide an initial explanation for the improvement in the presence of MiL construct observed in our sample group. Future studies using a longitudinal design should attempt to further investigate this issue.

As for our explored third point, i.e., to assess the influence of the search for MiL construct on the relationship between the presence of MiL construct and SI, we did not find that the search for MiL construct moderated the relationship as hypothesized. This novel finding reinforced, along with our first hypothesis, that the two constructs are independent of one another.

In summary, this study provided formal support for the role of the presence of MiL construct against SB in a general psychiatric study group. In contrast, the search for MiL construct was not related to SB and did not moderate the relationship between the presence of MiL construct and SI, suggesting, along with other findings, that the two constructs are independent of one another. This is the first study to examine the direct impacts of both constructs on SB in a clinical psychiatric population. To confirm these findings, our conclusions must be replicated in psychiatric clinical populations in settings other than a psychiatric ED and by using a longitudinal prospective and case-control study design.

This study had several limitations to consider. First, the use of regression analyses on cross-sectional data limited the interpretation of causality. Thus, this aspect should be interpreted with caution and replicated in longitudinal research. Second, we lacked a control group which limited the validity of our conclusions. Third, the MLQ questionnaire has not been formally validated in French. Fourth, the sample of patients we tested attended the psychiatric ED for SI and SA, and our results should be confirmed in suicidal patients belonging to more specialized psychiatric settings in order to be more generalizable. Finally, we did not test the impact of the two constructs using a typological methodology: the identification of different MiL profiles deriving from combinations of the two constructs can represent an opportunity to further refine individual diagnostic profiles with the goal of improving clinical outcomes by providing a more personalized approach.

## Data Availability Statement

The datasets generated for this study are available on request to the corresponding author.

## Ethics Statement

The protocol was approved by the Research Ethics Committee of Geneva under the registration number 14-168. All subjects gave written informed consent in accordance with the Declaration of Helsinki.

## Author Contributions

ACo drafted the primary manuscript, contributed to the conceptualization of the study, and participated to data collection and data interpretation. MB contributed to the conceptualization of the study, participated to data collection and data interpretation, and made statistical analysis. HR-L contributed to the conceptualization of the study and participated to study data collection. KW contributed to the conceptualization of the study and participated to data collection and data interpretation. MP and ACa contributed to the conceptualization of the study, corrected the manuscript, supervised all the steps of the work, and provided the intellectual impetus. All authors approved the final manuscript.

## Conflict of Interest

The authors declare that the research was conducted in the absence of any commercial or financial relationships that could be construed as a potential conflict of interest.
